# A rare case of retroperitoneal recurrence as squamous cell carcinoma 10 years after nephroureterectomy

**DOI:** 10.1002/iju5.12809

**Published:** 2024-11-04

**Authors:** Koichiro Uehara, Tatsuaki Onuki, Yukari Ishibashi, Sayuki Matsunuma, Hiroaki Ishida, Jiro Kumagai, Takayuki Murakami

**Affiliations:** ^1^ Department of Urology Yokohama City Minato Red Cross Hospital Yokohama Kanagawa Japan; ^2^ Department of Pathology Yokohama City Minato Red Cross Hospital Yokohama Kanagawa Japan

**Keywords:** pembrolizumab, retroperitoneal recurrence, squamous differentiation, upper tract urothelial carcinoma, urothelial carcinoma

## Abstract

**Introduction:**

Local recurrence for upper tract urothelial carcinoma typically occurs within 2 years post‐surgery. We report a rare case of retroperitoneal recurrence as squamous cell carcinoma 10 years after nephroureterectomy.

**Case presentation:**

A 67‐year‐old female was referred to our urology department for a left ureteral tumor. The surgical specimen of the laparoscopic left nephroureterectomy revealed urothelial carcinoma at the pT3 stage. Ten years post‐nephroureterectomy, magnetic resonance cholangiopancreatography revealed a mass lesion in the left retroperitoneum, a computed tomography‐guided biopsy revealed squamous cell carcinoma. Despite suspected distant metastases of other organ tumors, examinations such as digestive endoscopy and bronchoscopy did not reveal any tumor lesions. The patient was diagnosed with recurrent invasive urothelial carcinoma as a pathological feature of squamous cell carcinoma.

**Conclusion:**

The decision‐making process for treating malignant tumors, such as in cases with recurrence as squamous cell carcinoma, can be challenging.

**Keynote message**

Recurrence of renal pelvic ureteral cancer is usually observed within a few years of surgery, and recurrence 10 years after surgery is rare, as in this case. Furthermore, cases showing a change in the pathological tissue to squamous cell carcinoma of recurrence 10 years after surgery were not found within the scope of our investigation. The clinical decision‐making process for such patients is therefore challenging.

**Abbreviations & Acronyms**
CF: cisplatin/5‐fluorouracil
CT: computed tomography
FDG: fluorodeoxyglucose
H&E: hematoxylin and eosin
MRCP: magnetic resonance cholangiopancreatography
PD: progressive disease
PET: positron emission tomography
PR: partial response
PUC: pure urothelial cancer
SCC: squamous cell carcinoma
SqD: squamous cell differentiation
UC: urothelial carcinoma
UTUC: upper tract urothelial carcinoma
VUC: variant urothelial carcinoma

## Introduction

Local recurrence after surgery for UTUC varies in recurrence rates depending on risk factors, with recurrences usually occurring within 2 years after surgery. Local recurrence 10 years postoperatively is extremely rare. Herein, we report a rare case of retroperitoneal recurrence as SCC 10 years after nephroureterectomy.

## Case presentation

**Fig. 1:**
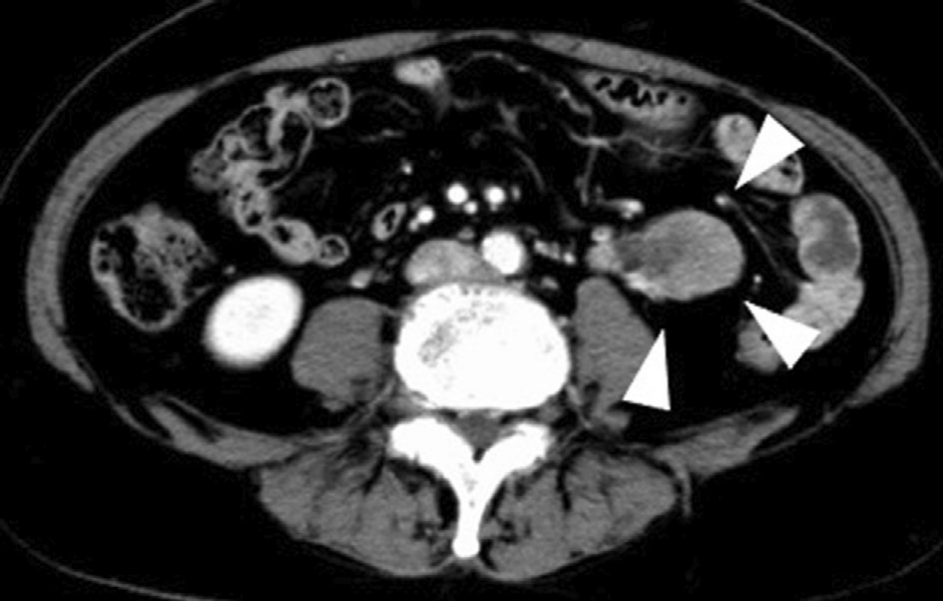
Pre‐treatment imaging. Abdominal contrast‐enhanced CT revealed a left ureteral tumor.

**Fig. 2:**
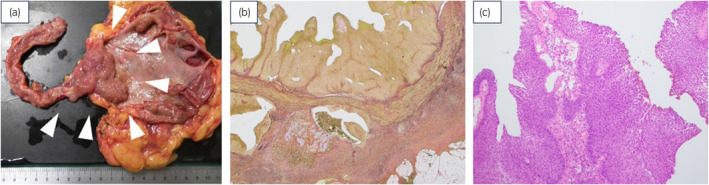
Gross and microscopic findings of the renal and ureteral tumors. (a) Lesions extending from renal pelvis to ureter. (b) UC showing papillary structures with parenchymal invasion (Elastica Van Gieson staining, ×20). (c) Stratified cellular structures with stratification centered on the stroma (H&E staining, ×100).

A 67‐year‐old female was referred to our urology department with a left ureteral tumor detected on CT. Contrast‐enhanced CT confirmed the diagnosis of left renal pelvic and ureteral cancer (cT3N0M0) (Fig. 1). A laparoscopic left nephroureterectomy was performed. The pathological diagnosis revealed UC at the pT3 stage, with the tumor extending from the renal pelvis to the lower ureter, although with negative surgical margins (Fig. 2a,b). No cellular component in this lesion displayed differentiation into squamous epithelium (Fig. 2c). Pathological examination revealed a Grade 3 component with a disorganized nuclear arrangement and increased cell density, nuclear chromatin, and nuclear fission pattern. There was lymphovascular invasion, no vascular invasion, and negative margins (pT3 Invasive UC Grade2>3 INFb ly1 v0 RM0). Subsequently, she received six courses of adjuvant chemotherapy with gemcitabine/nedaplatin therapy instead of gemcitabine/cisplatin therapy because of decreased renal function, and no subsequent evidence of recurrence was noted.

**Fig. 3:**
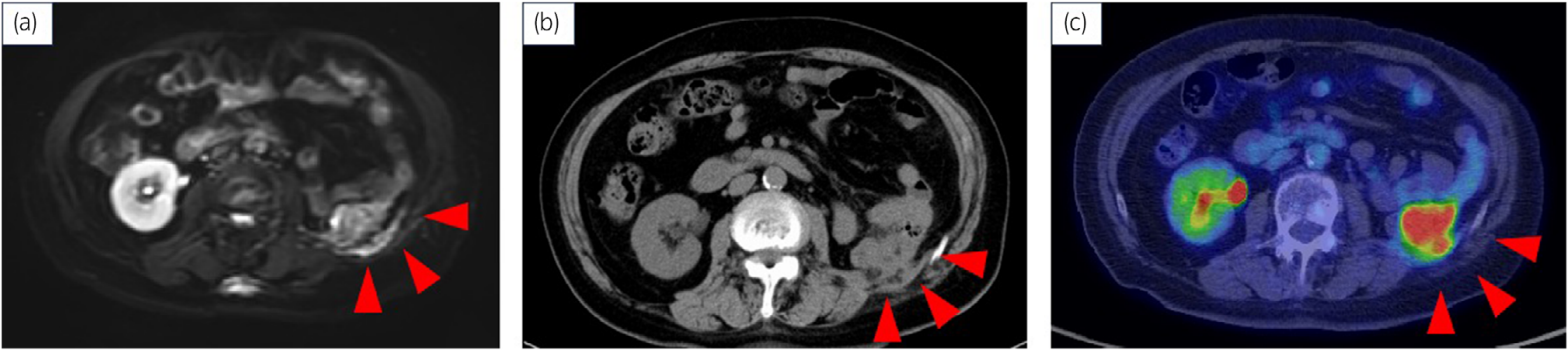
Post‐recurrence imaging. (a) MRCP showed a peritoneal lesion (diffusion weighted imaging). (b, c) PET‐CT showing FDG uptake in the peritoneal lesions.

**Fig. 4:**
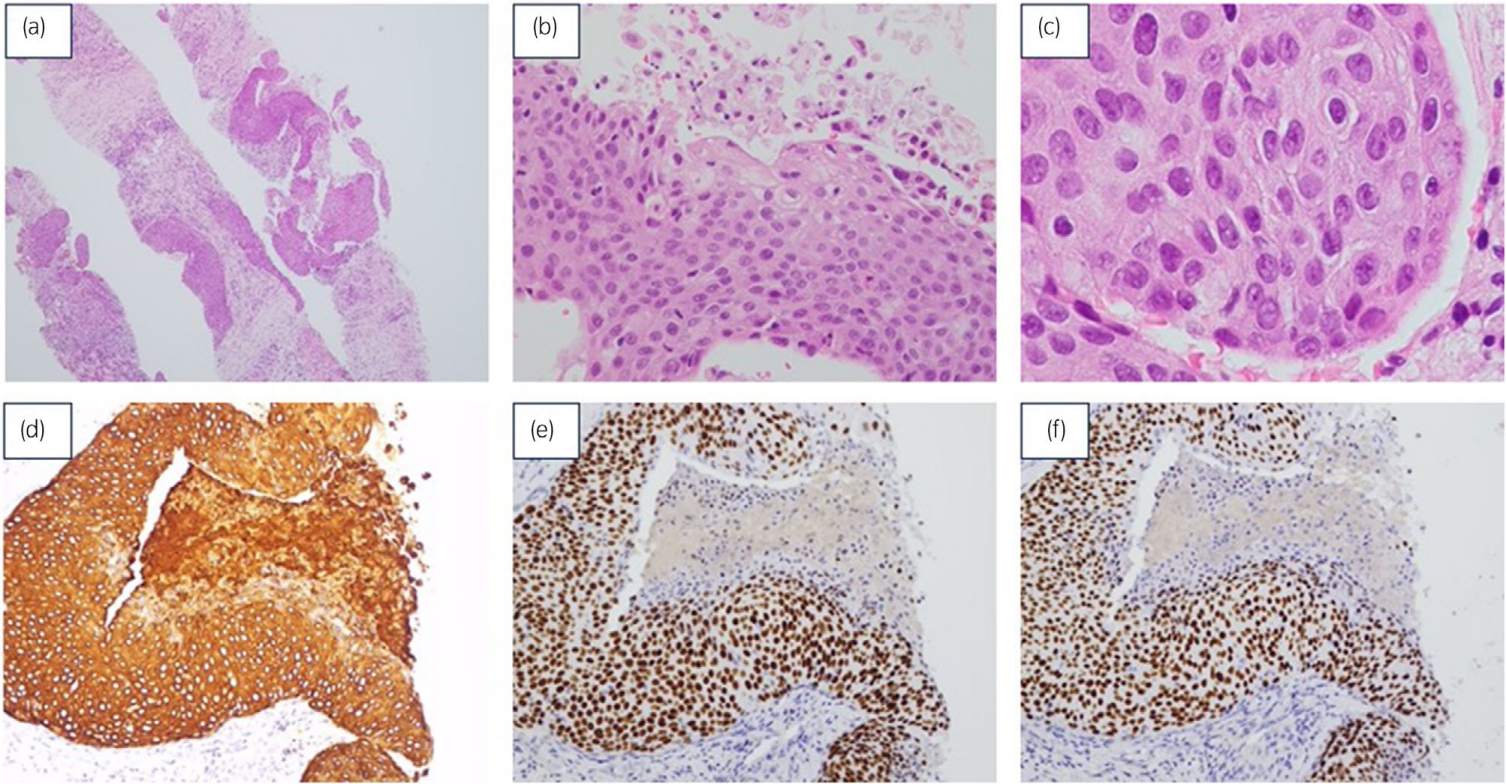
Microscopic findings of the peritoneal lesion (CT guided biopsy). (a) Tumor tissue with abundant fibrous components (H&E staining, ×20). (b) SCC with keratinization (H&E staining, ×400). (c) SCC with intercellular bridges (H&E staining, ×1000). (d–f) Pathological findings are positive for cytokeratin5/6 (d), P40 (e), and P63 (f).

Then, 10 years after nephroureterectomy, a MRCP imaging incidentally revealed a mass lesion in the left retroperitoneum (Fig. 3a), and a PET‐CT scan showed abnormal FDG uptake at the same site (SUV_max_ = 12.08) (Fig. 3b,c). A CT‐guided biopsy was performed for diagnosis. The histology of the retroperitoneal recurrent lesion showed SCC with keratinization and intercellular bridges (Fig. 4a–c). Immunohistochemical staining was diffusely positive for cytokeratin 5/6, P40 and P63 (Fig. 4d–f). There was no typical UC component in this lesion, as those observed in renal pelvis tumors.

Blood biochemical findings were as follows: white blood cell, 7200/mm^3^; hemoglobin (Hb), 13.3 g/dL; platelet, 22.4 × 10^4^/mm^3^; blood urea nitrogen, 16.5 mg/dL; creatinine, 1.01 mg/dL; aspartate aminotransferase, 23 IU/L; alanine aminotransferase, 13 IU/L; alkaline phosphatase, 102 IU/L;  lactate dehydrogenase, 229 IU/L; HbA1c, 8.4% (normal range: <6.3%); SCC, 39.2 ng/mL (normal range: <1.5 ng/mL); alpha‐fetoprotein, 8.65 ng/mL; and protein induced by vitamine K absence or antagonist II, 24 mAU/mL. [Correction added on 11 November 2024, after first online publication: The term ‘aspartate aminotransrerase’ has been corrected to ‘aspartate aminotransferase’.

Despite the suspected distant metastasis of other organ tumors, examinations such as digestive endoscopy and bronchoscopy did not reveal any tumor lesions. The patient was diagnosed with recurrent invasive UC as a pathological feature of SCC, and CF therapy was initiated. After two courses, the assessment showed an increase in retroperitoneal metastatic lesions, leading to the determination of PD. Consequently, pembrolizumab therapy was administered as the second‐line treatment, maintaining a PR for 11 months and causing a decrease in SCC to 2.5 ng/mL.

## Discussion

We encountered a rare case of retroperitoneal recurrence as SCC 10 years after nephroureterectomy. Local recurrence after surgery for UTUC varies depending on the risk factors (tumor in both the renal pelvis and ureter, T stage >2, lymph node involvement, grade 3 histology, and positive surgical margins), with recurrence usually occurring within 2 years of surgery.^1^ Local recurrence 10 years postoperatively is extremely rare.

Approximately 5–10% of UTUC exhibit SqD,^2^ which is closely associated with chronic irritation, infection, and inflammation.^3^ In the present case, the pathological diagnosis after nephroureterectomy was PUC without variants; however, the histological subtype at the time of recurrence differed from the initial diagnosis of renal pelvic and ureteral cancer. No lesions suggestive of a primary tumor were found, and considering that it was a retroperitoneal recurrence, it was diagnosed as a recurrence of renal pelvic and ureteral cancer. No local recurrence pattern showing a different histological appearance from PUC more than 10 years after surgery was found within the scope of our literature search. However, it is unlikely that an original PUC would recur as pure SCC 10 years later. Therefore, this case is more likely a UC recurrence with SqD.

Standard drug therapy for SCC of the urothelium has not been established^2^, ^4^, and only prospective studies of combination chemotherapy (ITP therapy; paclitaxel, ifosfamide, and cisplatin) have been reported.^5^ In addition, pembrolizumab therapy has been reported to be highly effective in the treatment of VUC.^6^ Other reports have suggested that the response of VUC to treatment with pembrolizumab was not inferior to that of PUC.^7^ In particular, the presence of SqD did not affect the response after pembrolizumab as compared with PUC or non‐squamous VUC.^8^ In this case, following the standard treatment for SCC in other areas,^9^ the patient underwent CF therapy, resulting in a PD assessment, but then received pembrolizumab therapy, maintaining PR.

There are no confirmed reports regarding the effectiveness of radiotherapy for local recurrence after nephroureterectomy.^1^ Considering the proximity of the recurrence site to the intestine, radiotherapy was not performed in this case.

Moving forward, continued follow‐up with CT scans and tumor markers will be conducted. If the efficacy of pembrolizumab therapy diminishes, paclitaxel will be used as the next therapy in accordance with the standard treatment for SCC in other areas.

## Conclusion

We encountered a rare case of peritoneal recurrence as SCC 10 years after a total nephroureterectomy. A recurrent pattern showing a different histological appearance as SCC more than 10 years after surgery has not been reported in the literature.

## Author contributions

Koichiro Uehara: Writing – original draft. Tatsuaki Onuki: Writing – review and editing. Yukari Ishibashi: Data curation. Sayuki Matsunuma: Data curation. Hiroaki Ishida: Data curation. Jiro Kumagai: Writing – review and editing. Takayuki Murakami: Supervision.

## Conflict of interest

The authors declare no conflict of interest.

## Approval of the research protocol by an Institutional Reviewer Board

Not applicable.

## Informed consent

Written informed consent for publication was obtained from the patient.

## Registry and the Registration No. of the study/trial

Not applicable.

## References

[1] Gao RW , Tollefson MK , Thompson RH *et al*. Predictors of locoregional recurrence and delineation of adjuvant radiation therapy fields for patients with upper tract urothelial carcinoma receiving nephroureterectomy. Pract. Radiat. Oncol. 2021; 11: e468–e476. 10.1016/j.prro.2021.02.005 33636378

[2] Rouprêt M , Babjuk M , Burger M *et al*. European Association of Urology guidelines on upper urinary tract urothelial carcinoma: 2020 update. Eur. Urol. 2021; 79: 62–79. 10.1016/j.eururo.2020.05.042 32593530

[3] Bhandari A , Alassi O , Rogers C , MacLennan GT . Squamous cell carcinoma of the renal pelvis. J. Urol. 2010; 183: 2023–2024. 10.1016/j.juro.2010.02.2370 20303510

[4] Flaig TW , Spiess PE , Agarwal N *et al*. Bladder cancer, version 3.2020, NCCN clinical practice guidelines in oncology. J. Natl. Compr. Cancer Netw. 2020; 18: 329–354. 10.6004/jnccn.2020.0011 32135513

[5] Galsky MD , Iasonos A , Mironov S *et al*. Prospective trial of ifosfamide, paclitaxel, and cisplatin in patients with advanced non‐transitional cell carcinoma of the urothelial tract. Urology 2007; 69: 255–259. 10.1016/j.urology.2006.10.029 17320659

[6] Bellmunt J , de Wit R , Vaughn DJ *et al*. Pembrolizumab as second‐line therapy for advanced urothelial carcinoma. N. Engl. J. Med. 2017; 376: 1015–1026. 10.1056/NEJMoa1613683 28212060 PMC5635424

[7] Minato A , Furubayashi N , Harada M *et al*. Efficacy of pembrolizumab in patients with variant urothelial carcinoma: a multicenter retrospective study. Clin. Genitourin. Cancer 2022; 20: 499.e1–499.e8. 10.1016/j.clgc.2022.05.001 35624001

[8] Kobayashi M , Narita S , Matsui Y *et al*. Impact of histological variants on outcomes in patients with urothelial carcinoma treated with pembrolizumab: a propensity score matching analysis. BJU Int. 2022; 130: 226–234. 10.1111/bju.15510 34110696

[9] Kitagawa Y , Ishihara R , Ishikawa H *et al*. Esophageal cancer practice guidelines 2022 edited by the Japan esophageal society: part 1. Esophagus 2023; 20: 343–372. 10.1007/s10388-023-00993-2 36933136 PMC10024303

